# Physiological properties of the visual system in the Green Weaver ant, *Oecophylla smaragdina*

**DOI:** 10.1007/s00359-023-01629-7

**Published:** 2023-04-13

**Authors:** Yuri Ogawa, Lochlan Jones, Laura A. Ryan, Simon K. A. Robson, Nathan S. Hart, Ajay Narendra

**Affiliations:** 1 School of Natural Sciences, Macquarie University, Sydney, NSW 2109 Australia; 2 Flinders Health and Medical Research Institute, Flinders University, Adelaide, SA 5001 Australia; 3 College of Marine and Environmental Sciences, James Cook University, Townsville, QLD 4814 Australia; 4 College of Science and Sustainability, CQ University Australia, Townsville, QLD 4812 Australia

**Keywords:** Spatial resolving power, Spatial acuity, Contrast sensitivity, Compound eye, Vision

## Abstract

The Green Weaver ants, *Oecophylla smaragdina* are iconic animals known for their extreme cooperative behaviour where they bridge gaps by linking to each other to build living chains. They are visually oriented animals, build chains towards closer targets, use celestial compass cues for navigation and are visual predators. Here, we describe their visual sensory capacity. The major workers of *O. smaragdina* have more ommatidia (804) in each eye compared to minor workers (508), but the facet diameters are comparable between both castes. We measured the impulse responses of the compound eye and found their response duration (42 ms) was similar to that seen in other slow-moving ants. We determined the flicker fusion frequency of the compound eye at the brightest light intensity to be 132 Hz, which is relatively fast for a walking insect suggesting the visual system is well suited for a diurnal lifestyle. Using pattern-electroretinography we identified the compound eye has a spatial resolving power of 0.5 cycles deg^−1^ and reached peak contrast sensitivity of 2.9 (35% Michelson contrast threshold) at 0.05 cycles deg^−1^. We discuss the relationship of spatial resolution and contrast sensitivity, with number of ommatidia and size of the lens.

## Introduction

Visually guided behaviours in ants have been studied in exclusively solitary foraging ants. This includes the scavenging desert ants (*Cataglyphis* (Wehner 2019), *Melophorus* (Narendra 2007) and *Ocymyrmex* (Müller and Wehner 2010)), predatory ants ((*Myrmecia* (Kamhi et al. 2020; Narendra et al. 2013a), *Diacamma* (Mukhopadhyay and Annagiri 2021)*, Odontomachus* (Rodrigues and Oliveira 2014)*, Paltothyreus* (Hölldobler 1980))*,* and non-specialist ants ((*Formica* (Woodgate et al. 2021), *Camponotus* (Schultheiss et al. 2015) *and Polyrhachis* (Narendra et al. 2013b))*.* Few studies have also investigated visual guidance in trail following ants ((*Iridomyrmex* (Card et al. 2016)*, Paraponera* (Harrison et al. 1989)*, Temnothorax* (Pratt et al. 2001)). Not surprisingly the visual systems and visual physiology is known only for some of these ants. The weaver ants of the genus *Oecophylla* are an iconic group of ants that has captured attention of naturalists (Hingston 1923) and evolutionary biologists (Hölldobler and Wilson 1990) due to their intricate social organisation and ecological success. Weaver ants are an exclusively Old-World genus, known by two extant species: *Oecophylla longinoda* from tropical Africa, and *Oecophylla smaragdina* found in the tropical and subtropical regions of India, South-east Asia and Australia (Hölldobler 1983; Hölldobler and Wilson 1990; Crozier et al. 2010). These ants are well-known for their extreme degree of cooperation and altruistic behaviour. This is evident in the arboreal colonies they establish in tree canopies where they bind leaves using silk from their own larvae (Hölldobler and Wilson 1990; Cole and Jones 1948). When neighbouring leaves are farther than an ants’ body length, they form living chains where individual ants seize each other’s petiole and pull together to bring the leaf margins closer (Hingston 1923). When two objects are not close enough for individual ants to cross, they form living chains to cross gaps. When ants have a choice, they choose to build bridges towards the closest structure (Hölldobler and Wilson 1978). This was the first indication that vision plays a role in these ants. Jander and Jander (Jander and Jander 1998) showed that *O. smaragdina* derive compass information from the sun and can orient to external artificial light sources. In the absence of celestial cues, weaver ants use the terrestrial landmarks to derive compass information.

**Fig. 1:**
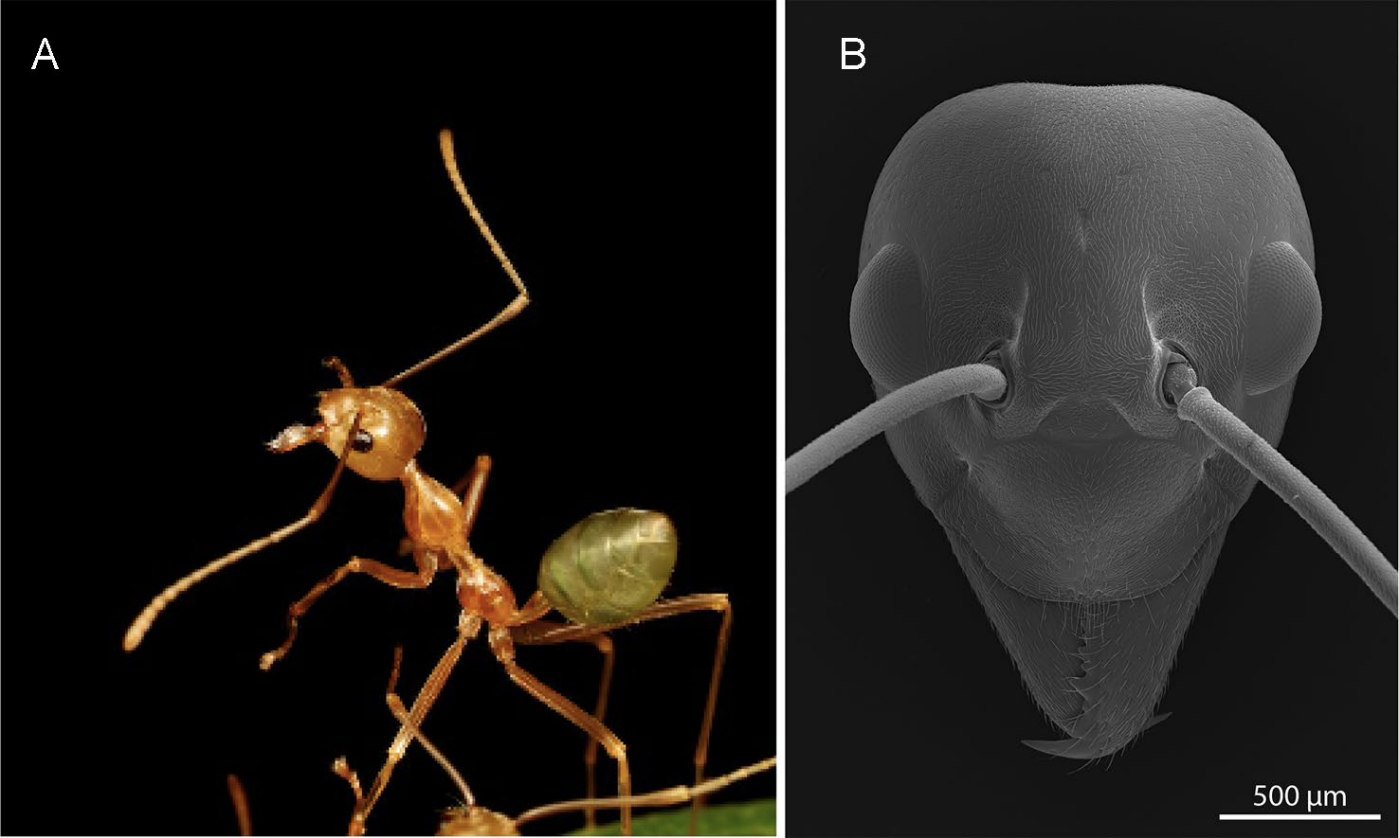
Worker of the Green Weaver Ant, *Oecophylla smaragdina,* Townsville, Australia. **A** A worker patrols the arboreal nest. **B** Scanning Electron Micrograph of the dorsal view of the head illustrates the size and location of the compound eyes. Image credits: Panel A: Ajay Narendra; Panel B: Sue Lindsay

In addition to relying on vision for navigation, individual weaver ants use vision to capture prey and while patrolling their arboreal nests (Hingston 1924). Given their reliance on visual information, it is essential to understand the visual capability of the compound eye of the weaver ant, *O. smaragdina*. Like most ant species, workers of *O. smaragdina* lack simple eyes (Fig. 1)*.* The visual capability of an eye is characterised by its temporal and spatial resolving power as well as contrast sensitivity, which is the ability to discriminate small objects in the visual scene and to differentiate them as their contrast decreases (Land 1997).

Typically, visual systems of fast moving and diurnal animals have faster phototransduction and higher temporal resolution compared to slowly moving and nocturnal animals (Boström et al. 2017; Frank 1999; Fritsches et al. 2005; Healy et al. 2013; Jenssen and Swenson 1974; Ryan et al. 2017; Warrington et al. 2017). Increasing the integration time of photoreceptors, for instance, improves photon capture, signal to noise ratio, and contrast discrimination (Frederiksen et al. 2008). This is analogous to having a longer shutter speed in a camera: a longer visual integration time makes the world brighter and improves the reliability of images in dim light (Nørgaard et al. 2008; Narendra et al. 2013c). But this comes at the expense of temporal resolution, which makes it difficult to perceive spatial detail while moving (Srinivasan and Bernard 1975), and fast-moving objects will appear blurry (Warrant 1999). Fast-moving ants such as *Pseudomyrmex phyllophilus*, have a photoreceptor impulse response duration of approximately 15 ms compared to the slow moving *Camponotus rufipes* and *Atta sexdens rubropilosa* (40–46 ms) (de Souza and Ventura 1989). The flicker fusion frequency (FFF) measures the ability of an eye to respond to a flickering light. Among ants, to the best of our knowledge, FFF has been measured only in *Myrmecia* species which invest significantly into vision (Narendra et al. 2017; Ogawa et al. 2022). The nocturnal *Myrmecia* tend to have lower FFF’s compared to their diurnal relatives (day-active: 142–189 Hz; night-active: 72–125 Hz).

The pattern electroretinography technique [pERG; (Porciatti 2007)] allows us to determine both the spatial resolving power and contrast sensitivity simultaneously. This may provide a more reliable estimate of spatial resolving power than anatomical methods especially for species in which behavioural estimates are difficult or time consuming to obtain (Ryan et al. 2017). Using this technique we have shown that the spatial resolving power of the nocturnal *Myrmecia midas* was 0.57 cycles per degree (cpd), and the diurnal *Myrmecia tarsata* was 0.60 cpd (Ogawa et al. 2019). The variation in the spatial resolving power was associated to ommatidial facet diameters, which were larger in the nocturnal *M. midas*. Interestingly, the contrast sensitivity functions did not differ between diurnal and nocturnal ants.

Here we used the same pERG technique to measure the spatial resolving power and the contrast sensitivity of the compound eye in workers of *O. smaragdina*. In addition, we determined the temporal resolution by recording the flicker fusion frequencies (FFF), the fastest flickering light an animal can still perceive as flickering at various light intensities. The critical FFF (cFFF) was determined using the highest light intensity. We also demonstrated the response properties of the compound eye by stimulating with a brief flash of light to analyse the impulse responses.

## Materials and methods

### Study species

We studied the visual system of workers of the day-active weaver ant *Oecophylla smaragdina* Fabricius (Fig. 1). Animals were caught from multiple nests at James Cook University campus, Townsville, Queensland (19.3179° S, 146.7288° E). Live animals were brought to Macquarie University, Sydney, NSW where both morphometric and physiological measurements were carried out. Research on ants does not require ethics approval in Australia.

### Morphometrics

Workers of this species exhibit distinct variation in body size with readily identifiable major and minor workers. We measured facet number, facet diameter and identified their relationship with head width in major and minor workers. Head width is often used as a body-size proxy in ants (Kaspari and Weiser 1999; Narendra et al. 2011). We took photographs of the dorsal surface of the heads of 30 minor and 44 major workers using a digital camera (D5600 DSLR, Nikon) and measured the widest part of their head using ImageJ (National Institutes of Health, Bethesda, MD, US).

To measure the facet number and diameter of each ant, we prepared eye replicas using established techniques. Detailed methods are described elsewhere (Ramirez-Esquivel et al. 2017). Each eye replica was photographed with a digital camera (Olympus DP21, Olympus Australia, Victoria) attached to a light microscope (Olympus BX40, Olympus Australia, Victoria). The total number of facets was counted in all individuals. Diameter of eighty facets (representing approximately 10% of total facets) from the entire eye was measured in five minor workers and six major workers.

### Electroretinography (ERG)

The temporal characteristics of *O. smaragdina* (*n* = 5) were determined by measuring the impulse response and the flicker fusion frequency (FFF) using electroretinography (ERG). Electrophysiological methods used were described earlier (Ogawa et al. 2022). Briefly, animals were immobilised on ice for 5–10 min before removing their antennae and legs. Each individual ant was fixed, dorsal side up, to a plastic stage with bees’ wax before being mounted in a Faraday cage. A platinum wire of 0.127 mm diameter was inserted into the mesosoma and served as the indifferent electrode. As an active electrode, another platinum wire of 0.127 mm diameter was placed against the lateral surface of the compound eye with conductive gel (Livingstone International Pty Ltd., New South Wales, Australia). ERGs were recorded through a differential amplifier (× 1000; DAM50, World Precision Instruments Inc., FL, USA), with high- and low-pass hardware filter cut-off frequencies of 0.1 and 1 k Hz respectively, connected to a computer via a data acquisition unit (Micro1401-3, Cambridge Electronic Design Ltd., Cambridge, England).

Light stimulus was provided to the frontal area of the compound eye with a 5 mm diameter cool white light emitting diode (LED; C503C-WAS-CBADA151, Cree Inc, Durham, NC, USA). The angular size of LED was two degrees from the animal’s perspective that was set at 14 cm from the animal at 10° elevation. All experiments were performed at room temperature (21–25 °C) in the dark. Animals were dark-adapted for 20 min before each experiment. We carried out these experiments during the day (0900–1700 h), to ensure it aligns with diurnal activity rhythm that these ants exhibit.

The amplitude of the impulse response was measured as the voltage responses to a 1 ms square wave flash of light followed by 2 s of darkness. The response was averaged over 100 repetitions. The light source produced the maximum irradiance of (5.81 × 10^–5^ W/cm^2^) at the surface of the eye (ILT1700, International Light Technologies). To identify the temporal characteristics of the impulse response we measured the following parameters at the highest light intensity, peak amplitude (mV), response latency (ms), time to peak amplitude (ms) and response duration (ms). The *peak response amplitude* was measured as the maximum amplitude of hyperpolarizing response of the eye. Response *latency* was defined as the time taken for the response to exceed 3 standard deviations of noise after stimulus onset. The standard deviation of the noise was calculated from all voltage changes in the last 500 ms before stimulus onset. *Time to peak amplitude* was taken as the time of stimulus onset to the response peak. The *duration of the impulse response* was measured as the full-width of the response at half the maximum amplitude.

The FFF was estimated as the highest temporal frequency at which the ERG reached a criterion threshold. The experimental design has been described in detail in previous studies (Ogawa et al. 2022; Warrington et al. 2017). Briefly, the visual stimulus followed a square-wave flicker over a range of stimulation frequencies from 2 to 200 Hz. Each frequency was presented for 20 s and the average response amplitude calculated using a Fast Fourier Transform. FFFs were measured at 11 different light levels over a 5-log unit intensity range (1.33 × 10^–9^ to 5.81 × 10^–5^ W/cm^2^), increasing in 0.5 log unit steps apart from the lowest stimulus intensity (relative intensity at 0.00002). To evaluate any degradation of the response over time, the FFF at the highest intensity was tested before starting the series of FFF measures with 20 min dark adaptation in between. The LED generated an electric artefact that seemed like the response of the eye. The largest possible artefact was measured as the maximum signal amplitude recorded at the highest light intensity by covering the LED with a black cloth and then used as the response threshold. FFF was defined as the frequency at which the response power (log10 of the response amplitude power) crossed the threshold for each animal (see Fig. 1 in Warrington et al. 2017).

### Pattern electroretinography (pERG)

Pattern electroretinograms were used to assess the spatial resolving power and contrast sensitivity of *O. smaragdina* (*n* = 3). Detailed methods are described previously (Palavalli-Nettimi et al. 2019; Ogawa et al. 2019; Ryan et al. 2020). Briefly, animals were fixed on a plastic stage as described above and we used the same set of electrodes. Responses were amplified by a differential amplifier and sent to a 16-bit analog-to-digital converter device (USB-6353, National Instruments, Austin, TX, USA) connected to a computer. Individual animals were placed 30 cm from a white screen (51 cm width × 81 cm height). The screen displayed vertical contrast-reversing sinusoidal gratings of different spatial frequencies and Michelson’s contrasts (Michelson 1927), projected by a digital light processing projector (W1210ST, BenQ corporation, Taipei, Taiwan).

The stimuli were generated using Psychtoolbox 3 (Pelli 1997). The mean irradiance of the grating stimuli was 1.75 × 10^–4^ W/cm^2^ measured using a calibrated radiometer (ILT1700, International Light Technologies, Peabody, MA, US). A temporal frequency of 2 Hz was used for all stimuli.

Prior to initiating recordings, a uniform grey stimulus with the same mean irradiance as the grating stimuli was presented to the ants for 20 min. They were then presented with 11 spatial frequencies (0.58, 0.53, 0.47, 0.42, 0.37, 0.32, 0.26, 0.21, 0.16, 0.11, 0.05 cpd), and up to five contrasts (95%, 75, 50, 25, 12.5) with the same mean irradiance for each spatial frequency. To control any degradation of the response over time, the spatial frequencies of the gratings were presented in the order of decreasing frequencies of every second spatial frequency. The interleaved spatial frequencies were then presented in ascending order. At each spatial frequency, all five different contrasts were tested in decreasing order. For each combination of the stimuli, the response for five seconds each was recorded fifteen times to average them in the time domain. The averaged responses were then analysed using a Fast Fourier Transform, FFT in the frequency domain. The non-visual electric signal (i.e., background noise) was measured as a control at two spatial frequencies (0.1 and 0.05 cpd) at 95% contrast with a black board used to shield the ant from the visual stimuli before and after the experimental series. The maximum signal out of the four control runs was used as the noise threshold.

For each eye that we carried out pERG recordings, we prepared eye replicas to determine the total facet number and diameter of 30 facets arbitrarily selected from the medio-frontal region. We also measured the head width of these ants.

### Estimation of spatial resolving power and contrast threshold

An *F* test was used to assess whether the response signal at the second harmonic (4 Hz) of the FFT response spectrum differed significantly from ten neighbouring frequencies, five on either side, for each spatial frequency and contrast combination. Spatial resolving power and contrast threshold were obtained by interpolating from the last point above the noise threshold whose amplitude at 4 Hz was also significantly greater than the ten surrounding frequencies, and the first point below the noise threshold. If the first point below the noise threshold was not significantly greater than the ten surrounding frequencies, the last point above the threshold was considered as the spatial resolving power, without interpolating between two data points. Contrast sensitivity is defined as the inverse of contrast threshold.

#### Statistical analysis

We determined the relationship between facet numbers, head width and worker caste. We assessed this with a linear mixed model using the maximum likelihood (ML) estimation method implemented in the nlme package of RStudio (Version 1.1.419, RStudio, Inc. Boston, MA, US). Head width and worker caste and their interaction were used as fixed effects in the model. Animal identity was used as a random effect.

To assess the relationship between facet diameter and worker castes, we used a linear mixed model. Worker caste was used as a fixed effect and animal identity as a random effect in the model. A linear mixed effects model was used for testing whether the FFF differed according to stimulation light intensities. Stimulation light intensity was used as a fixed effect and animal identity was used as a random effect. We used a linear model to evaluate any degradation of the response over time by comparing the first FFF at the brightest light, which was recorded prior to a series of various light intensities, to the last recording of FFF at the same light intensity.

## Results

### Relationship between eye size and body size

**Fig. 2:**
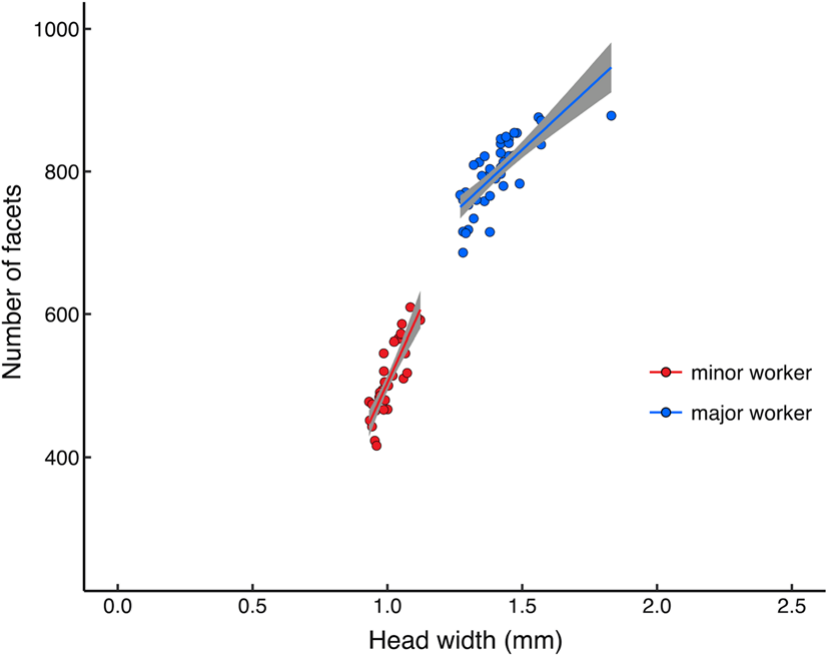
The relationship between head width and number of facets in each eye among workers of *Oecophylla smaragdina*. Best fit linear regression lines are included for major and minor workers. 95% confidence interval is shown in grey. Sample number: minor worker = 30; major worker = 44

We studied two distinct castes of *O. smaragdina*: minor workers with a head width of 1.0 ± 0.05 mm (*n* = 30, mean ± s.e.m) and major workers with a head-width of 1.42 ± 0.11 mm (*n* = 44) (Fig. 2, Table 1). The number of ommatidia in each compound eye varied between the two castes (Table 2). Minor workers had 508.2 ± 9.50 ommatidia, whereas major workers had 804.2 ± 7.67 ommatidia (mean ± s.e.m). The facet diameter did not vary between minor and major workers (minor workers: 17.26 ± 0.18 µm (mean ± s.e.m); *n* = 5; 80 facets; major workers: 16.98 ± 0.2 µm, n = 6; 80 facets, Table 3).

**Table 1:** Spatial resolution, head width, and eye measurements of *Oecophylla smaragdina* workers

	Minor worker	Major worker
Head width (mm)	1.0 ± 0.05	1.42 ± 0.11
Facet number per eye	508.2 ± 9.5	804.2 ± 7.67
Facet diameter (µm)	17.26 ± 0.18	16.98 ± 0.2
Facet diameter in the medio-frontal region (µm)		17.72 ± 0.09
cFFF (Hz)		132 ± 3
Spatial resolving power (cpd)		0.52 ± 0.03
Maximum contrast sensitivity at 0.05 cpd		2.88 ± 1.12 (34.67%)

**Table 2:** Results of the linear mixed model analysis for testing the relationship between number of facets and castes of worker and head width

Terms (added or subtracted from final model)	df	logLik	L. Ratio	*P* value
**Caste (minor or major)**	4	− 395.56	164.26	< 0.001
**Head width**	5	− 363.63	228.11	< 0.001
**Caste: head width**	6	− 355.06	17.15	< 0.001

Final model: Number of facets ~ caste + head width + caste: head width + (1|animal ID). Bold terms had a significant effect and used in the final model

**Table 3:** Results of the linear mixed model analysis for testing the relationship between facet diameter and castes of worker

Terms (added or subtracted from final model)	df	logLik	L. Ratio	*P* value
Caste (minor or major)	4	− 1497.24	1.21	0.27

Final model: Number of facets ~ 1 + (1|animal ID)

For electrophysiological investigations, we studied major workers that had head width of 1.44 ± 0.05 mm (mean ± s.e.m, *n* = 5), 784.0 ± 5.0 ommatidia in each eye and the diameter of individual facet in the medio-frontal region was 17.72 ± 0.09 µm (mean ± s.e.m, *n * = 5). These numbers aligned well with the larger dataset shown in Table 1.

### Impulse response

**Fig. 3:**
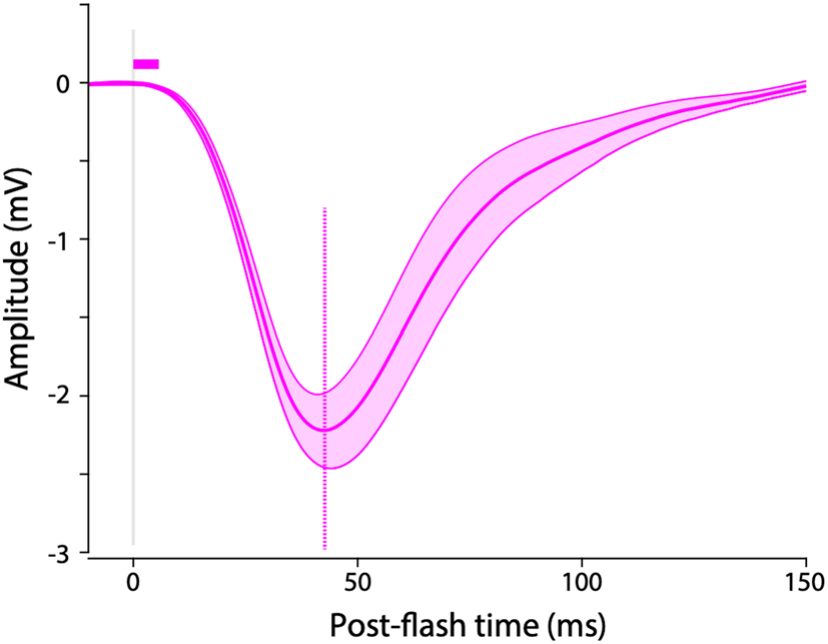
Averaged impulse response curve in workers of *Oecophylla smaragdina* (mean ± s.e.m, *n* = 5). Horizontal solid line above the curves indicates the latency. Dashed vertical line indicates the time to peak amplitude. A grey vertical line indicates the flash timing

We recorded stable and repeatable electroretinograms (ERGs) from major workers during the day. The response amplitude to 1 ms flash of brightest light was measured in five *O. smaragdina* major workers (Fig. 3). Peak response amplitude was − 2.33 ± 0.27 mV (mean ± s.e.m, *n* = 5). The latency of the response was 4.64 ± 2.37 ms. The time taken to reach the peak response amplitude was 42.56 ± 2.95 ms. The duration of responses was 42.32 ± 3.41 ms (Table 4)

**Table 4:** Results of the linear mixed model analysis for testing the relationship between FFFs and intensity of light

Terms (added or subtracted from final model)	df	logLik	L. Ratio	*P* value
**Intensity of light**	13	− 156.25	243.38	< 0.001

Final model: FFFs ~ intensity of light + (1|animal ID). Bold term had a significant effect and used in the final model

### Temporal resolution

**Fig. 4:**
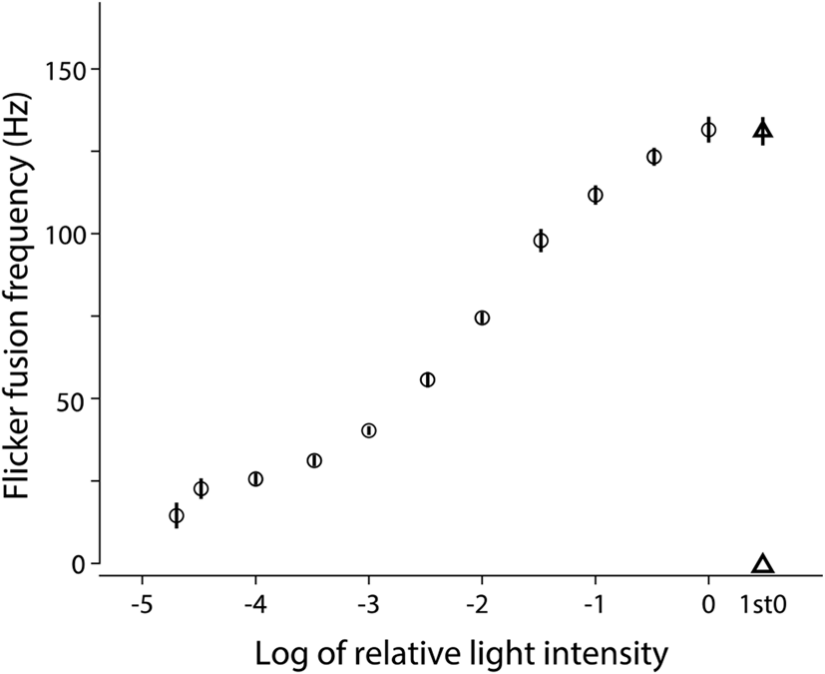
Flicker fusion frequency (FFF) at different light intensities in workers of *Oecophylla smaragdina* (mean ± s.e.m., *n* = 5). Prior to a sequence of measurements at increasing light intensities, the FFF was first measured at the highest light intensity (a triangle marker) to verify the stability of the recording over long durations of time. There was no difference between the first and the last measurements of FFF at the highest light intensity

The temporal resolution was determined by measuring the flicker fusion frequency (FFF) across a sequential range of increasing light intensities (Fig. 4). The FFF increased with light intensity and the maximum FFF for the brightest light stimulus was 132 ± 3 Hz (mean ± s.e.m, *n* = 5). It was not significantly different from a measurement of 131 ± 4 Hz recorded at the same light intensity prior to a series of light intensities to evaluate any degradation of the response over time (*R*^2^ = 0.00077, F_(1, 8)_ = 0.006181, *p* value = 0.94) (Table 4).

### Spatial resolving power and contrast sensitivity

Pattern electroretinography (pERG) was used to investigate the spatial resolving power and contrast sensitivity in *O. smaragdina*.

**Fig. 5:**
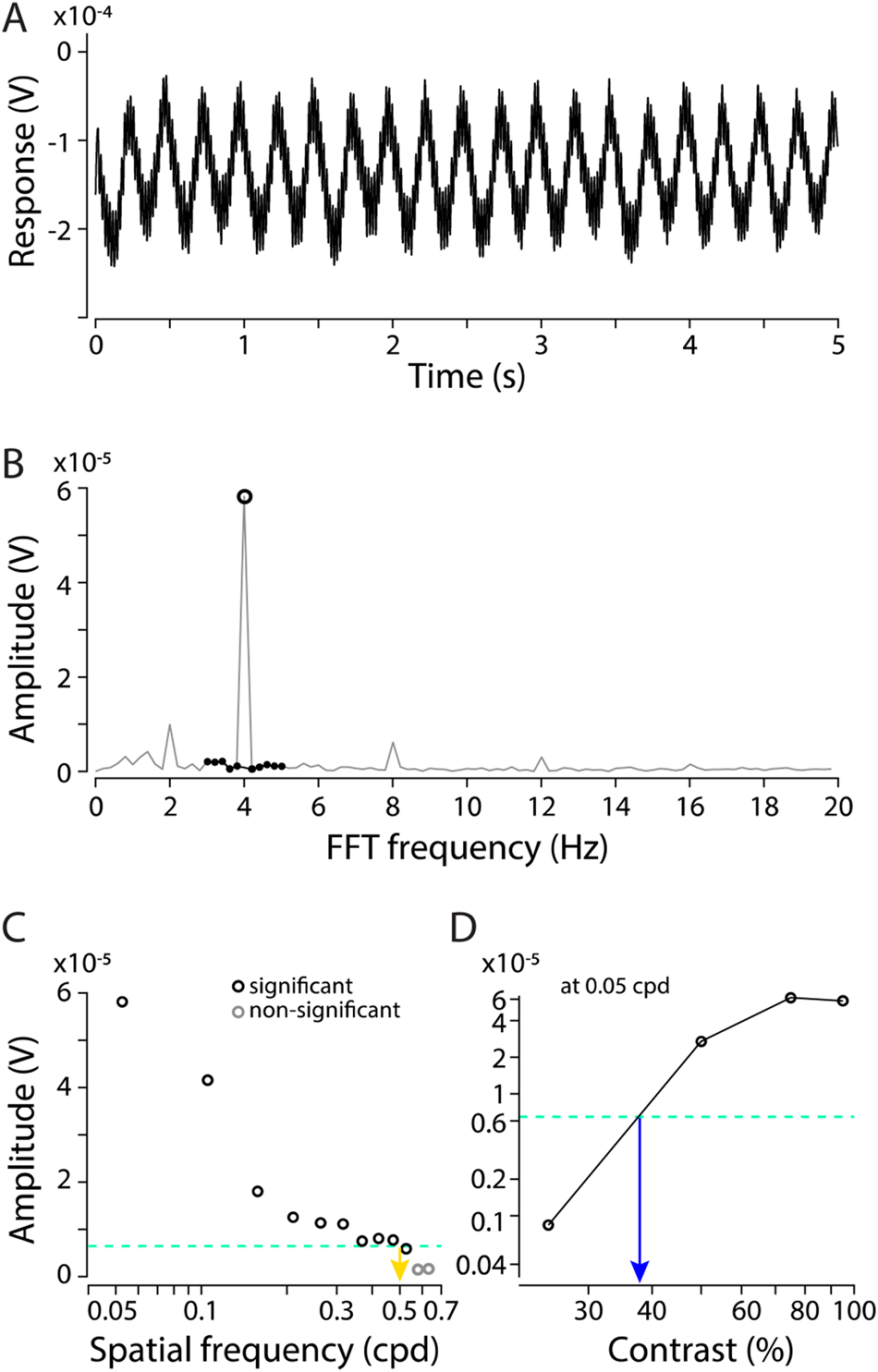
Pattern electroretinogram (pERG) measurements in *Oecophylla smaragdina*. **A** Raw trace of a measurement from the compound eye of an ant, in response to the pattern-reversal sinusoidal gratings of temporal frequency 2 Hz with spatial frequency of 0.05 cpd and 100% contrast. This trace is a mean of 15 repeats of 5 s measurements. The response amplitude changes in a wave manner as it oscillated at the reversal changes of gratings, changing from dark to bright at 2 Hz. **B** FFT analysis shows the response amplitude peaking at the second-harmonic frequency (4 Hz). The response signal at 4 Hz (open circle) is assessed with F-test whether it is significantly different from ten neighbouring frequencies, five on either side (black dots). **C** Responses to 95% contrast gratings as a function of spatial frequency. Spatial resolving power is obtained by interpolating from the last point above and the first point below a noise threshold (green dash line), which is the maximum signal from control treatments where the ant was shielded from the stimuli. Black data points indicate significant peaks in the voltage signal at 4 Hz; the grey data point indicates that the peak value was not significantly different from ten neighbouring frequencies. **D** Contrast threshold, the lowest contrast to which the eye could respond, shown for one spatial frequency of the stimuli. Spatial resolving power and contrast threshold are the x-axis values at the intersection of the dashed lines in C and D, respectively. Contrast sensitivity is defined as the inverse of contrast threshold

The amplitude of the pERG response at the second harmonic of the stimulus modulation frequency decreased with increasing spatial frequency or decreasing contrast of the visual stimuli (Fig. 5). Spatial resolving power was 0.52 ± 0.03 cpd (mean ± s.e.m., *n* = 3) in *O. smaragdina*.

**Fig. 6:**
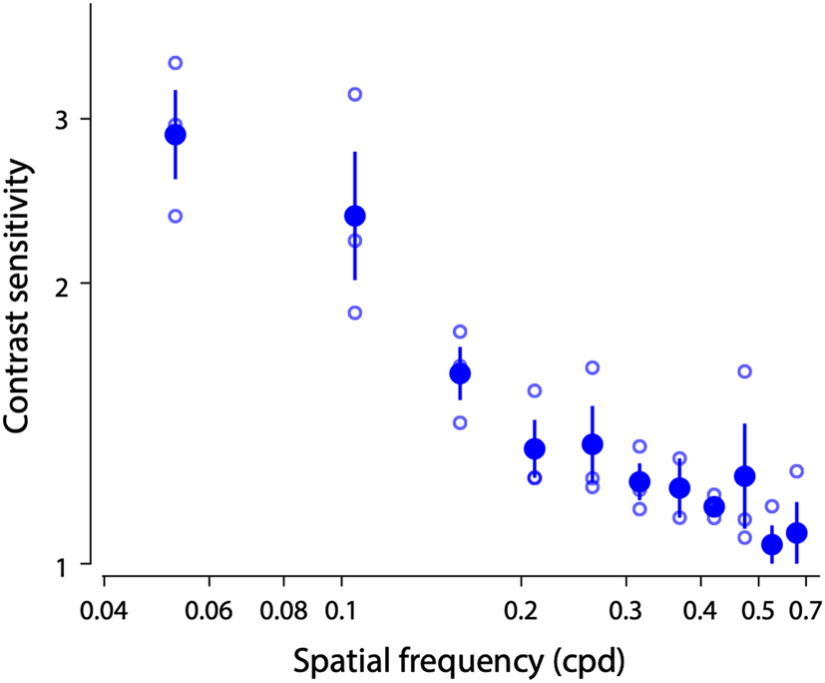
Contrast sensitivity function for *Oecophylla smaragdina* obtained from pattern electroretinogram (pERG) measurements. Data are means ± 95% confidence intervals of contrast sensitivity (1/contrast threshold) measured from three individuals. Individual measurements are shown as open blue circles

The contrast threshold was lower at lower spatial frequency (0.05 cpd) and increased at higher spatial frequencies (Fig. 6). No contrast threshold was recorded for the highest spatial frequency (0.62 cpd) because responses for that frequency never reached the threshold. Contrast thresholds for all spatial frequencies were used to calculate the contrast sensitivities (1/contrast threshold) shown in Fig. 6. The contrast sensitivity reached a maximum of 2.88 at 0.05 cpd in *O. smaragdina*.

## Discussion

The major workers in *O. smaragdina* had more ommatidia in each eye compared to minor workers, but the facet diameters were comparable in both castes. The facet diameter in major workers was slightly larger in the medio-frontal area of compound eye. We discuss here the physiology of the compound eye in *O. smaragdina* in comparison with other ants and insects.

### Impulse response

Four response properties of compound eyes, peak response amplitude, latency, time to peak response amplitude and duration of the impulse response, were measured by using a brief flash of light as a stimulus. The peak response amplitude to a 1 ms flash of light in *O. smaragdina* was approximately − 2.3 mV. It is comparable to that of − 2.7 mV measured in diurnal jack jumper ant *Myrmecia croslandi*, whose compound eye consists of about 2363 ommatidia with a facet diameter of 12 to 22 µm (Ogawa et al. 2022).

The temporal characteristics of impulse response in *O. smaragdina* suggests their eyes are well suited for a diurnal lifestyle. This is also suited for their slow-moving repertoire compared to the fast moving and jumping behaviour in *Myrmecia*. The impulse response duration in *O. smaragdina* (42.32 ms) is similar to the slow moving *Camponotus rufipes* and *Atta sexdens rubropilosa* (40—46 ms) in which intracellular recordings were carried out (de Souza and Ventura 1989). In contrast, the fast-moving *Pseudomyrmex phyllophilus* had a photoreceptor response duration of approximately 15 ms (de Souza and Ventura 1989). The longer duration of impulse response, which is the integration time of photoreceptors, is beneficial to enhance optical sensitivity, signal to noise ratio, and contrast discrimination (Frederiksen et al. 2008) for slow moving or nocturnal animals. The temporal characteristics of the visual system of *O. smaragdina* have thus evolved in accordance with their slow moving and day-active lifestyle (de Souza and Ventura 1989; Howard et al. 1984).

### Temporal resolution

The temporal resolution of major workers of *O. smaragdina* was assessed by measuring the flicker fusion frequencies (FFF) at various light intensities, with the maximum FFF measured with the brightest light intensity of stimuli, known as critical FFF (cFFF). Major workers of *O. smaragdina* had a cFFF of 132 Hz. This was close to that seen in day-active *Myrmecia* ants (142—189 Hz), but certainly faster than the cFFF of nocturnal *Myrmecia* ants (72—125 Hz) (Ogawa et al. 2022). The cFFF of *O. smaragdina* is slower compared to the day-active jack jumper ant, *M. croslandi* (cFFF: 189 Hz) that visually tracks and jumps to capture small flying insects (Ogawa et al. 2022). The relatively fast cFFF in *O. smaragdina* major workers therefore suggests that their visual system is well adapted for bright light condition. Fast diphasic responses with cFFF up to 300 Hz are found in flying insects (e.g., bees and flies) and slow monophasic responses with cFFF of 20 Hz are seen in locusts and crickets (Autrum 1958).

### Spatial resolving power

A comparison between *O. smaragdina* major workers to other ant species reveals a possible lower limit in the spatial resolving power in ants. Spatial resolving power in ants tend to decrease as facet numbers and facet diameter decreases (Table 5) (Palavalli-Nettimi et al. 2019). In contrast, though the number of facets in *O. smaragdina* (facet number: 804) was more than that seen in *Rhytidoponera inornata* (227) and *Polyrhachis nr. aurea* (522), all three ant species had comparable spatial resolving power (Palavalli-Nettimi et al. 2019). Spatial resolving power *O. smaragdina* was 0.52 cpd, *Rhytidoponera inornata* (0.48 cpd) and *Polyrhachis nr. aurea* (0.51 cpd). Among these three species, *O. smaragdina* had the largest facet diameters (17.7 µm) compared to *R. inornata* (12.8 µm) and *P. nr. aurea* (12.5 µm). It thus appears that 0.5 cpd could be a minimal spatial resolving power to ensure their compound eye can support visually guided behaviour.

**Table 5:** Summary of current knowledge of visual physiology in ants

Species	Number of facets	Facet diameter	cFFF (Hz)	Spatial acuity (cpd)	Maximum contrast sensitivity at 0.05 cpd
^*1*^Oecophylla smaragdina*	804.2 ± 7.67	16.98 ± 0.2	132 ± 3	0.52 ± 0.03	2.88 ± 1.12 (34.67%)
^*2*^*Myrmecia croslandi*^*2*^	2363	22	188.7 ± 3.93	–	–
^*3*^*Myrmecia nigrocincta*	2483 ± 42	20.5 ± 0.5	–	0.52 ± 0.0005	20.68 ± 0.6 (4.8%)
^*2,3*^*Myrmecia tarsata*	2627 ± 120	22.40 ± 0.40	154.2 ± 6.6	0.60 ± 0.01	15.51 ± 0.7 (6.4%)
^*2,3*^*Myrmecia midas*	3590 ± 88	31.62 ± 0.47	84.6 ± 3.2	0.57 ± 0.01	21.2 (4.7%)

*major workers only

^1^this article; ^2^(Ogawa et al. 2022); ^3^(Ogawa et al. 2019)

### Contrast sensitivity

Contrast sensitivity is the ability to discriminate pattern as their brightness contrast decreases (O'Carroll and Wiederman 2014). The sensitivity of a compound eye depends on the overall ommatidia number and the size of the individual facets (Horridge 1977). In *O. smaragdina* we found that the maximum contrast sensitivity reached to 2.9 (34.67% Michelson’s contrast) at 0.05 cpd, which was lower compared to *M. tarsata* and *M. midas* (Table 5), but higher than *P. nr. aurea* (2.2) and *R. inornata* (1.3) (Palavalli-Nettimi et al. 2019). In *Myrmecia* ants, though *M. midas* had larger facets compared to *M. tarsata* (Table 5), facet size did not explain the variation seen in contrast sensitivity in these two species (Ogawa et al. 2019). Along similar lines, though facet diameters *O. smaragdina* (17.7 µm) were larger compared to *R. inornata* (12.8 µm) and *P. nr. aurea* (12.5 µm), facet size is unlikely to explain the difference in their contrast sensitivity. Though smaller facets limit the amount of incident light, a number of other factors can influence the sensitivity of the eye. This includes ommatidial *F* number, rhabdom diameter (Kirschfeld 1974; Land 1981; Frederiksen and Warrant 208), photoreceptor properties (Laughlin and Weckström 1993; Frederiksen et al. 2008) and neural summation strategies (Ribi WA 1975; Greiner et al. 2004; Stöckl et al. 2016) which are yet to be determined in these ants.

Based on their visual physiology, workers of *O. smaragdina* can be classified as slow-moving ants and their visual system is well suited to a diurnal lifestyle, which aligns with their known behaviours. Their temporal resolution further affirms their diurnal lifestyle. Their eyes are however slightly slower than the jack jumper ants that visually track and hunt prey. Now that we have a clear understanding of their physiological visual capabilities it sets the scene to investigate the individual behavioural capabilities of these weaver ants.

## Data Availability

Complete dataset is available here: https://tinyurl.com/34tsf244.
